# Assessing core, e-learning, clinical and technology readiness to integrate telemedicine at public health facilities in Uganda: a health facility – based survey

**DOI:** 10.1186/s12913-019-4057-6

**Published:** 2019-04-29

**Authors:** Vincent Micheal Kiberu, Richard E. Scott, Maurice Mars

**Affiliations:** 1 Department of TeleHealth, Nelson R. Mandela School of Medicine, University of KwaZulu–Natal, Durban, South Africa; 2 NT Consulting – Global e-Health Inc, Calgary, Alberta Canada; 3 University of Calgary, Calgary, Alberta Canada

**Keywords:** Telemedicine, Uganda, E-readiness, Health facilities

## Abstract

**Background:**

In developing countries like Uganda, there are shortages of health workers especially medical specialists. The referral process is frustrating to both patients and health workers (HWs). This is due to delays in accessing laboratory results/tests, costs of travel with resultant delay in consulting specialists. Telemedicine can help reduce these problems. To facilitate successful and sustainable telemedicine implementation the eHealth readiness of different stakeholders should be undertaken. This study was conducted at public health facilities (HFs) in Uganda to assess eHealth readiness across four domains; core, e-learning, clinical and technology, that might hamper adoption and integration of telemedicine.

**Methods:**

A cross-sectional study using mixed methods for data collection was conducted at health center IVs, regional and national referral hospitals. The study was conducted in three parts. Quantitative data on core, e-learning and clinical readiness domains were collected from doctors and other healthcare providers (nurses/midwives, public health officers and allied healthcare workers). Respondents were categorised into ‘aware and used telemedicine’, ‘aware and not used’, ‘unaware of telemedicine’. Focus Group Discussions were conducted with patients to further assess core readiness. Technology readiness was assessed using a questionnaire with purposively selected respondents; directors, heads of medical sections, and hospital managers/superintendents. Descriptive statistics and correlations were performed using Spearman’s rank order test for relationship between technology readiness variables at the HFs.

**Results:**

70% of health professionals surveyed across three levels of HF were aware of telemedicine and 41% had used telemedicine. However, over 40% of HWs at HC-IV and RRH were unaware of telemedicine. All doctors who had used telemedicine were impressed with it. Telemedicine users and non-users who were aware of telemedicine showed core, clinical, and learning readiness. Patients were aware of telemedicine but identified barriers to its use. A weak but positive correlation existed between the different variables in technology readiness.

**Conclusion:**

Respondents who were aware of and used telemedicine across all HF levels indicated core, learning and clinical readiness for adoption and integration of telemedicine at the public HFs in Uganda, although patients noted potential barriers that might need attention. In terms of technology readiness, gaps still exit at the various HF levels.

**Electronic supplementary material:**

The online version of this article (10.1186/s12913-019-4057-6) contains supplementary material, which is available to authorized users.

## Background

Telemedicine is the remote delivery of clinical and other healthcare services using information and communications technologies (ICT). Health professionals in developed countries use telemedicine to broaden communication with patients and fellow professionals. Indeed, the American Telemedicine Association states "Telehealth has advanced from a curious form of clinical communication to a mainstay in the way providers and consumers interact" [1]. It is anticipated that telemedicine will have a more profound impact in developing countries than in developed countries [2] but successful implementation largely depends on the ‘buy-in’ of local people as recommended by Zilgalvis and Jungmann, [3, 4].

The World Health Organization (WHO) estimates the world will be short of 12.9 million healthcare workers (HWs) by 2035; today, that figure stands at 7.2 million. There is an extreme shortage of HWs in 57 countries, 63% of which are in sub-Saharan Africa [5]. Interventions like task shifting after training have been adopted but HWs in developing countries have continued to experience challenges in communication and consultation with peers [6]. In the African Region of the WHO, there are only 27 doctors per 100,000 people compared to 321 per 100,000 people in Europe [7]. The shortage of doctors and especially specialists is more acute in rural areas [8]. The referral process and subsequent consultation is frustrating for both patients and HWs due to long waiting times, delays accessing laboratory results, duplication of laboratory tests, the cost of travel, and the time taken to consult specialists [9]. Telemedicine can help address these problems by providing timely access to specialist and other healthcare, reducing the need and associated costs of travel, reducing consultation waiting time, and promoting home-based care [10].

eHealth readiness is defined as the “preparedness of healthcare institutions or communities for the anticipated change brought by programs related to information and communications technology” [11]. A lack of readiness has led to failure of eHealth implementations [12]. At least eight types of eHealth readiness have been described [13]. A recent review of eHealth readiness frameworks suggested a rank order of readiness themes based on the frequency of their occurrence in the literature analysed: Technological readiness, core/need/motivational readiness, acceptance and use readiness, organisational readiness, IT skills/training/learning readiness, engagement readiness, and societal readiness [14]. The lack of consistency in terms, content, and definitions applied suggests readiness aspects of importance to a specific setting must be considered. In this paper the focus is on four eHealth readiness domains of importance to Uganda: core, clinical, e-learning (electronic learning), and technology readiness.

Core readiness has been defined as the extent to which members of a community are dissatisfied with the current status of their healthcare service provision, see eHealth as a solution, and express their need and preparedness for eHealth services [13]. Clinical readiness refers to the readiness to provide clinical telemedicine services, as well as the ability to identify any gaps within an existing clinical telemedicine programme [15]. e-Learning means a mode of instruction intended to support learning, but delivered via a digital device such as a computer or mobile device, therefore, e-learning readiness refers to an institution’s, or an individual’s, readiness to adopt this mode of instruction and delivery [16].

Finally, regarding technology readiness, Rezai-Rad et al. [17] identified that this is associated with several components. These included the availability and affordability of ICT resources (e.g., hardware and software), quality of ICT infrastructure, use of networks, availability of ICT technical personnel, and IT security. However, a convenient definition has been provided by Mauco et al.: Technology readiness “gauges the availability and affordability of ICT resources necessary to implement a proposed eHealth innovation (e.g., skilled human resources, ICT support, quality ICT infrastructure, and power supply)” [13].

In Uganda, the National Healthcare System is a combination of the public and private sectors. The public sector is made up of all health facilities (HFs) owned by the government of Uganda, and supervised and regulated by the Ministry of Health. Other HFs include the health services of the Ministries of Defence, Education, Internal Affairs (the Police and Prisons), and Ministry of Local Government. The private health sector consists of private not-for-profit providers, private health practitioners, as well as traditional and complementary medicine practitioners. Provision of healthcare services by public HFs is decentralized at district and sub-district levels, each with a role in delivery and management of health services. The public healthcare system is structured into National Referral Hospitals (NRHs), Regional Referral Hospitals (RRHs), General Hospitals (GHs), and four levels of Health Centres (HC); HC-IV, HC-III, HC–II and Village Health Teams or HC-I.

There have been several initiatives by the Ugandan government to improve physical structure at district, regional and national referral HFs. However the healthcare system in Uganda still faces enormous challenges including insufficient technology resources, limited use of these resources (i.e. computers and Internet), and regular power blackouts. In addition, there are limited human resources (IT professionals) to provide technical support due to high staff turn-over and redeployment, a common occurrence in Uganda’s health sector [18]. Despite this, the use of eHealth, and of ICT for communication in and between HFs in Uganda, has recently been documented [19, 20]. For example, within the past 5–10 years, a variety of eHealth and telemedicine initiatives have been trialled. These include m-health tools such as WinSenga (a foetal heart rate monitor using a smart phone), Matibabu (a non-invasive malaria test), Text to Change (an SMS–based app to scale up HIV/AIDs awareness and promote HIV counselling and testing), a disease surveillance and medication tracking tool, a local EMR at an HIV/AIDs clinic, and videoconference-based exchanges with Universities in the USA for research, education and clinical practice [20]. However, no eHealth readiness assessment of core, clinical, e-learning or technology readiness has been conducted to assess challenges to telemedicine integration within Uganda’s healthcare system. This may explain in part why several eHealth innovations that have been trialled have not gone beyond the proof of concept or pilot stage [20].

This study evaluated four eHealth readiness domains (core, clinical, e-learning and technology) that might hamper adoption and integration of telemedicine services at HFs in Uganda. Prior review of the literature on eHealth readiness assessment frameworks had revealed these four domains were critical in assessing eHealth readiness in developing countries. Study results will help mitigate any future barriers when implementing telemedicine technologies at HFs in Uganda, and potentially elsewhere.

## Methods

This was a cross sectional study using mixed methods for data collection. Two semi-structured questionnaires were developed to collect quantitative data; first, for core, e-learning, and clinical readiness (Additional file 1), and second for technology readiness (Additional file 2). One health facility at the RRH and HC-IV levels was selected from each of three regions (Western, Northern and Eastern) using multi-stage random sampling. Mulago hospital, which functions as an HC-IV, GH and referral hospital for the Central region, was purposively identified for the study.

The technology readiness questionnaire was distributed to managers and physicians in-charge who were purposively selected. The core, e-learning, and clinical questionnaire was pre-tested for validity/consistency with 10 HWs at a HC-IV in Mukono district in the Central region of Uganda. It was then distributed to doctors, nurses/midwives, public health officers (PHOs), and allied healthcare workers at the different facilities who were purposively selected. The first part of the questionnaire addressed awareness and / or use of telemedicine. Respondents were divided into three groups. The ‘telemedicine user’ group was further categorised into two sub-groups, doctors, and other health professionals and administrators. The other two groups included those who were not aware of telemedicine (‘telemedicine naïve’ group) and those who were aware of telemedicine but had not used it (‘telemedicine aware’ group). Each group was analysed to assess core, e-learning and clinical readiness to integration of telemedicine.

The four eHealth readiness domains were assessed in 17 questions, core (10), e-learning (3), clinical (3) and overall readiness (1). The 10 core questions were further divided into readiness to integrate telemedicine into practice (4), comfort with telemedicine (3) and process workflow (3). The questions were either dichotomous yes/no responses or used a 5-point Likert scale (strongly agree to strongly disagree). The results are presented as positive responses with the strongly agree and agree combined. Responses were compared using Fishers exact test with alpha set at 5%. eHealth readiness was assessed based on quartiles of the average scores: 0–25% (no positive perception), 26–50% (a potentially positive perception requiring nurturing), 51–75% (a generally positive perception that can be leveraged), and 76–100% (a favourably positive perception that can be leveraged).

To further assess core readiness, Focus Group Discussions (FGDs) were conducted with patients at Out-Patient Departments (OPD) at the identified HFs (Additional file 3). Convenience sampling was used to select the OPD patients, with patients invited by word of mouth to participate in the study. Each FGD comprised six to eight participants with seven FGDs conducted. Written notes were made of the discussions because participants had reservations about audio recording. Contemporaneous notes from the moderator and an assistant were used to ensure key points of the discussion and quotes were correctly and accurately documented. The discussions were summarised and data coded according to major themes, and sub-themes identified.

The technology readiness questionnaire was used to collect quantitative data on technology readiness from purposively selected participants (Additional file 4). These included directors, heads of medical sections, and hospital managers/superintendents. Descriptive statistics and correlations using Spearman’s rank order test were performed. Alpha was set at 5%.

## Results

### Core, clinical and e-learning readiness Assessment

In total, 406 HWs responded to the core, clinical and e-learning readiness questionnaire (Table 1). The sample included more nurses and allied health workers than doctors.

**Table 1:** Demographic data of respondents by: health facility level, job category

	Nurses/ Midwives n (%)	Allied Health Workers n (%)	Public Health Officers n (%)	Doctors n (%)	Missing n (%)	Total N
HC-IV	39 (43.8)	24 (27.0)	22 (24.7)	4 (4.5)	0	89
RRH	55 (31.6)	45 (25.9)	29 (16.7)	44 (25.3)	1 (0.6)	174
NRH	22 (15.3)	46 (32.2)	26 (18.2)	49 (34.3)	0	143
Total	116 (28.5)	115 (28.3)	77 (19.0)	97 (23.9)	1 (0.3)	406

Respondents’ awareness of telemedicine and their use of telemedicine is shown in Table 2. Only 30.3% of people had not heard of telemedicine (telemedicine naïve group), while 41.1% were ‘telemedicine users’, of whom significantly more doctors 74 (76.3%) had used telemedicine than other health professionals (*p* < 0.0001). The remainder (28.6%) constituted the ‘telemedicine aware’ group.

**Table 2:** Awareness and use of telemedicine services

Health Facility	Telemedicine Naïve Group n (%)	Aware of Telemedicine but not used it n (%)	Aware of and used Telemedicine n (%)	
			Doctors	Others
HC IV (*n* = 89)	42 (47.1)	32 (36)	3 (3.4)	12 (13.5)
RRH (*n* = 174)	71 (40.8)	32 (18.4)	29 (16.7)	42 (24.1)
NRH (*n* = 143)	10 (7.0)	52 (36.4)	42 (29.3)	39 (27.3)
Total (*n* = 406)	123 (30.3)	116 (28.6)	74 (18.2)	93 (22.9)

The responses of doctors and other health professionals who have used telemedicine were further analysed according to several themes and subthemes identified in Table 3. A more detailed breakdown of these results by health facility is presented in Additional file 1.

**Table 3:** Analysis of doctors and others who have used telemedicine (TM)

Characteristics		TM Users			Non-users	
		Doctors (*n* = 74)	Others (*n* = 93)	Total (*n* = 167)	TM aware non-users (*n* = 116)	Unaware of TM (*n* = 123)
Core Readiness - Awareness of Telemedicine						
TM used for	Diagnosis	16 (34.0)	9 (9.7)	25 (15.3)^a^	–	–
	Treatment	23 (31.1)	14 (15.2)	37 (22.7)^a^	–	–
	Prevention	25 (33.8)	15 (16.1)	40 (24.5)^b^	–	–
	e-Learning	30 (40.5)	40 (43.0)	70 (42.9)	–	–
	Knowledge sharing	21 (28.4)	31 (33.3)	52 (31.9)	–	–
	e-consultation	47 (63.5)	46 (49.5)	93 (57.1)	–	–
	e-prescription	60 (81.1)	1 (1.1)	61 (37.4)^c^	–	–
Impressed with TM		74 (100)	81 (87.1)	155 (95.1)^c^	–	–
Core Readiness - Integrating Telemedicine						
TM reduces referrals		65 (87.8)	73 (78.5)	138 (84.7)	89 (76.7)	30 (24.4)^c^
TM reduces hospital visits		65 (87.8)	60 (64.5)	125 (76.7)^c^	99 (85.3)^a^	52 (42.3)^c^
Use TM over traditional methods		67 (90.5)	69 (74.2)	136 (83.4)^b^	102 (87.9)	67 (54.5)^c^
Required before TM	Licensing	43 (58.1)	64 (68.8)	107 (65.6)	85 (73.3)	48 (39.0)^c^
	Remuneration	33 (44.6)	52 (55.9)	85 (52.1)	61 (52.6)	20 (16.3)^c^
	Policy	74 (100)	80 (86.0)	154 (94.5)^c^	97 (83.6)^a^	58 (47.2)^c^
	Training	68 (91.9)	79 (84.9)	147 (90.2)	63 (54.3)^c^	66 (53.7)^c^
	Ethical Guideline	69 (93.2)	61 (65.6)	130 (79.8)^c^	66 (56.9)^c^	45 (36.6)^c^
TM solves HWs crisis		70 (94.6)	74 (79.6)	144 (88.3)^b^	106 (91.4)	81 (65.9)^c^
Core Readiness - Comfort with Telemedicine						
TM is cost effective		59 (79.7)	93 (100)	152 (93.3)^c^	98 (84.5)	85 (69.1)^c^
Worth investing in TM infrastructure?		73 (98.6)	76 (81.7)	149 (91.4)^c^	83 (71.6)^c^	81 (65.9)^c^
TM is effective for emergencies		66 (89.2)	86 (92.5)	152 (93.3)	111 (95.7)	96 (78.0)^b^
Core Readiness – Process Workflow						
TM affects normal process workflow		5 (6.8)	23 (24.7)	28 (17.2)^b^	5 (4.3)^c^	24 (19.5)
TM would alter work practices		24 (32.4)	47 (50.5)	71 (43.6)^a^	54 (46.6)	44 (35.8)
TM would change referral process		63 (85.1)	66 (71.0)	129 (79.1)^a^	113 (97.4)^c^	53 (43.1)^c^
Learning Readiness – Enhancing Provider Skill						
How do you access Continuing Medical Education?	Conference	71 (95.9)	65 (69.9)	136 (83.4)^c^	91 (78.4)	70 (60.3)^c^
	Internet	70 (94.6)	56 (60.2)	126 (77.3)^c^	83 (71.6)	45 (38.8)^c^
	Workshop	74 (100)	63 (67.7)	137 (84.0)^c^	93 (80.2)	86 (74.1)^a^
	Attend course	26 (35.1)	22 (23.7)	48 (29.4)	42 (36.2)	60 (51.7)^c^
	Consult colleagues	8 (10.8)	11 (11.8)	19 (11.7)	9 (7.8)	17 (14.7)
Could TM enhance e-learning?		62 (83.8)	62 (66.7)	124 (76.1)^a^	114 (98.3)^c^	113 (91.9)^c^
Are HW ready to adopt e-learning?		68 (91.9)	81 (87.1)	149 (91.4)	107 (92.2)	113 (91.9)
Would TM use bridge skills gap?		49 (66.2)	78 (83.9)	127 (77.9)^b^	104 (89.7)^b^	119 (96.7)^c^
Clinical Readiness - Factors						
Use of TM for clinical gaps		67 (90.5)	84 (90.3)	151 (92.6)	109 (94.0)	104 (84.6)
Agree with treatment outcome		70 (94.6)	85 (91.4)	155 (95.1)	111 (95.7)	99 (80.5)^b^
TM will improve clinical outcome		70 (94.6)	85 (91.4)	156 (95.1)	100 (86.2)	100 (81.3)^b^
Overall Readiness						
HW ready to integrate TM		65 (87.8)	60 (64.5)	125 (76.7)^b^	68 (58.6)^b^	64 (52.0)^c^

^a^ **≤ 0.05,** ^**b**^ **≤ 0.01,** ^**c**^ **≤ 0.001***.* Statistical comparison between doctors and others who use telemedicine is shown in the Telemedicine Total column, between users and those aware of telemedicine but not using it in Telemedicine Aware non-users column, and between telemedicine users and telemedicine naïve respondents in the Unaware of TM column

Attitudes of those who had used telemedicine were positive. Over 90% of telemedicine users felt that it was cost effective, improved clinical outcomes and considered it was worth investing in telemedicine infrastructure. Over 75% felt telemedicine reduced referrals, changed the referral process, reduced hospital visits, addressed the shortage of health workers, and were happy to use it in place of traditional methods. It was also seen as a means of facilitating education with 91% feeling that health workers were ready to adopt e-learning. Significantly fewer doctors felt that it adversely affected the normal workflow (*p* < 0.01) and altered work practices (*p* < 0.05) than other telemedicine users. The need for eHealth policy, guidelines and training was identified by more than 90% of doctors.

Significant differences were noted between doctors and other users with respect to their overall impression of telemedicine, in terms of cost effectiveness, reducing hospital visits, solving the health worker shortage, its impact on normal workflow and work processes, and the worth of investment in telemedicine infrastructure.

Significantly, more ‘telemedicine aware’ respondents felt that it would reduce hospital visits, change the referral process, bridge the skills gap, and enhance e-learning than telemedicine users. Fewer felt that it adversely affected the normal process workflow, was worth investing in telemedicine infrastructure, and that health workers were ready for telemedicine. The ‘telemedicine naïve’ group were generally sceptical about the value of telemedicine, its use, and investing in it, although they were positive about e-learning and its use in bridging the skills gap.

**Fig. 1:**
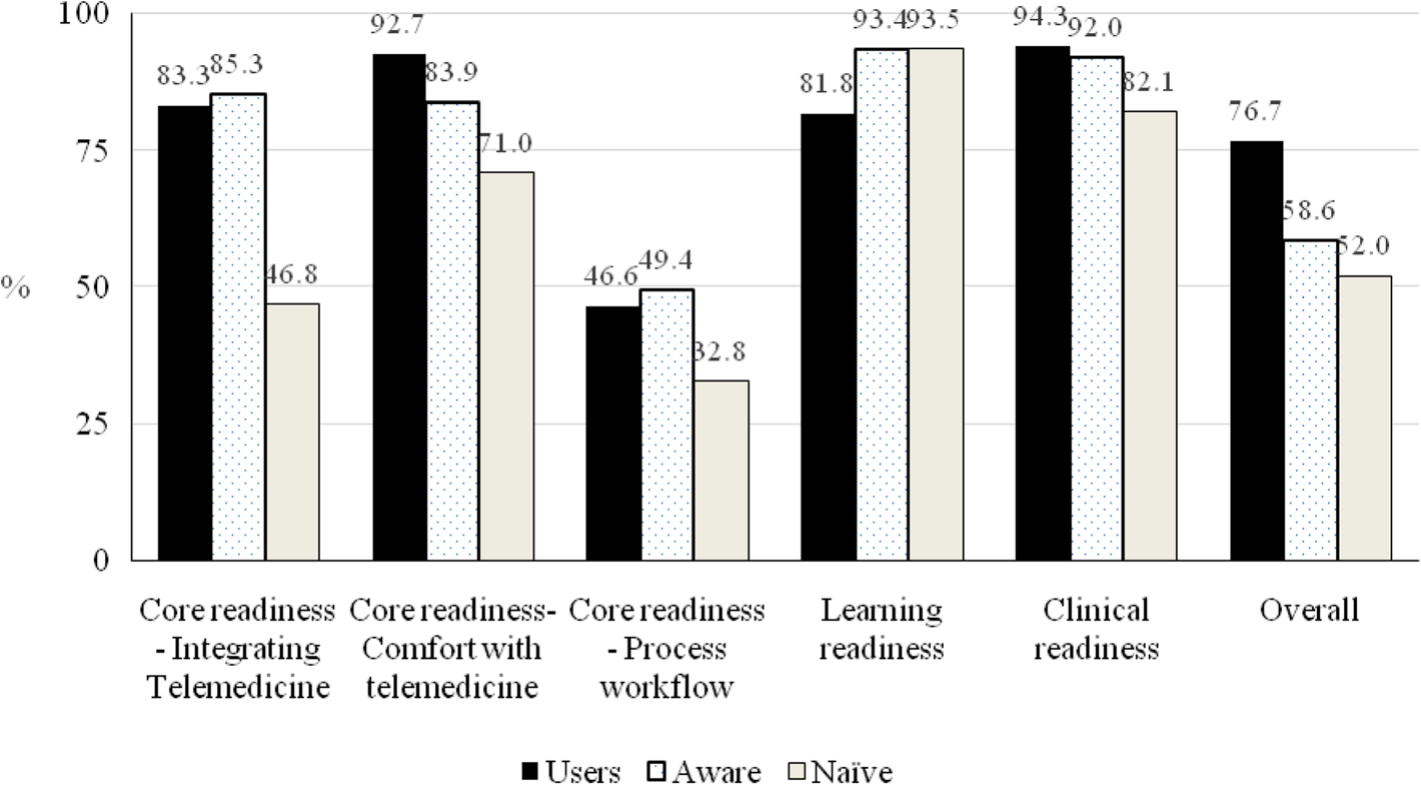
The average scores for core, clinical, e-learning and overall readiness of telemedicine users, telemedicine aware, and telemedicine naïve groups

The percentage of positive responses for each readiness domain was averaged for telemedicine users, telemedicine aware, and telemedicine naïve groups. Overall readiness was based on the single question about health workers’ readiness to integrate telemedicine into practice (Fig. 1).

The user and aware groups averaged over 80% positive responses for all but process workflow, a domain in which low scores for changes to work flow and practice indicated a positive attitude to telemedicine. However, they expressed less overall readiness. Over 80% of both telemedicine users and aware groups were positive about core readiness, and over 80% of all respondents were positive about clinical and e-learning readiness. As expected, telemedicine users expressed the most overall readiness for telemedicine.

### Patients’ perspective on Core readiness

#### Awareness of telemedicine

Participants from most of the FGDs, especially those at RRHs and NRHs, were aware of the concept of telemedicine. Some, particularly those at HC-IVs, said they had heard and were aware of the term ‘telemedicine’ but were not sure if they used it (telemedicine) at health facilities within their locale. In contrast those at higher health facility levels (RRH & NRH) were generally aware of telemedicine services (email and telephone) and had used them to communicate with medical workers/specialists:

“*We are aware about telemedicine where a doctor treats a patient at a far distance using technology. Not sure though if this can work in our health centres because in most cases you need to see the doctor/ nurse and properly explain your sickness.”* (HC-IV patient).

“*In most cases you call and/or email your doctor to consult him/her about your health condition and s/he responds with certain prescriptions, advice … ..we believe that’s telemedicine! We have used it though at times don’t realize it’s actually the telemedicine*.” (RRH patient).

“*We know what telemedicine is all about but different telemedicine services like mobile phone for calling, WhatsApp and email may work well in our hospital setting. Services like video conferencing can be used with hospitals in developed countries.”*(NRH patient).

#### Comfort using telemedicine

Participants explained shortcomings they had experienced while using telemedicine to seek and/or receive healthcare within their hospital settings.

“*Often times when you’re feeling unwell, you just walk to the health centre to see the doctor/nurse for treatment. Although some cases you can always call to make an appointment with a specific clinician/doctor but in all you need to have physical interaction before you can receive treatment.”*(HC-IV patient).

“*We have found it comforting and cost effective consulting and/or securing an appointment with the doctor. Experience has shown, what the doctor/clinician does, is to recommend First Aid treatment but also advise you to visit the nearest health centre. But if it’s a follow-up treatment the doctor may provide you with medical advice without necessarily asking you to come to the health centre*.” (RRH patient).

“*There [are] those emergencies when you can’t easily reach the health centre but only to call the doctor for the quickest solution. Such cases are possible if there’s prior physical interaction with the doctor otherwise you will always be referred to the nearby health centre. It’s not easy to explain your health details to a stranger unless if there is a formal arrangement with a health centre*.” (NRH patient).

#### Integrating telemedicine

Patients at all health facility levels were concerned about barriers to the integration of telemedicine within the public health sector in Uganda. Issues raised related to laboratory tests, prescriptions, records, and the cost of telemedicine.

“*When we use telemedicine to seek healthcare how then do we receive the drugs? How about if there’s need for a blood test and to keep my patient’s form? Those things (Telemedicine) can work well in developed countries*.” (HC-IV patient).

“*This might work for absentee/scarce health workers … maybe reducing congestions at hospitals … although challenges [exist] such as; heavy investment by government to train health workers, providing extra incentives, building the infrastructure, and subscribing to data and voice networks*.” (RRH patient).

### Technology readiness assessment

Overall, 128 respondents completed the technology readiness questionnaire. Respondents were from one HC-IV and RRH from each of the three regions, plus the NRH. Only the District Health Office in one district responded to the questionnaire. The distribution of the respondents is shown in Table 4, and responses to the questionnaire in Table 5.

**Table 4:** Demographic Data of the 128 Respondents by Health Facility level/Office, Job Title

Job Title	HCIV	RRH	NRH	DHO
District Health Officials	0	0	0	6 (4.7%)
Consultants	0	20 (42.6%)	15 (26.8%)	0
Heads of Section/ Units / Directors	16 (12.5%)	27 (57.4%)	41 (73.2%)	0
Hospital - In charge	3 (2.3%)	0	0	0
Total	19 (14.8%)	47 (36.7%)	56 (43.8%)	6 (4.7%)

**Table 5:** Summary statistics for the 128 respondents on technology readiness assessment

General Theme	Characteristics	Response	District 1		District 2		District 3			NRH (*N* = 56)
			HC-IV (*n* = 7)	RRH (*n* = 20)	HC-IV (*n* = 8)	RRH (*n* = 15)	HC-IV (*n* = 4)	DHO’s (*n* = 6)	RRH (*n* = 12)	–
General Question	Knowledge about TM	No	3 (42.9)	0	0	0	1 (25)	0	0	0
		Yes	4 (57.1)	20 (100)	8 (100)	15 (100)	3 (75)	6 (100)	12 (100)	56 (100)
		Missing	0	0	0	0	0	0	0	0
ICT Technical Personnel	Existence of ICT section at the HF.	No	6 (85.7)	0	8 (100)	0	0	0	0	0
		Yes	0	16 (80)	0	15 (100)	4 (100)	0	11 (91.7)	56 (100)
		Missing	1 (14.3)	4 (20)	0	0	0	6 (100)	1 (8.3)	0
	Access to ICT Expert?	No	0	14 (70)	8 (100)	0	4 (100)	0	8 (66.7)	0
		Yes	3 (42.9)	0	0	15 (100)	0	0	0	56 (100)
		Missing	4 (57.1)	6 (30)	0	0	0	6 (100)	4 (33.3)	0
	Access to ICT consultancy services	No	0	14 (70)	8 (100)	0	4 (100)	0	8 (66.7)	0
		Yes	3 (42.9)	0	0	15 (100)	0	0	0	56 (100)
		Missing	4 (57.1)	6 (30)	0	0	0	6 (100)	4 (33.3)	0
Existing ICT Equipment & Budget	Existence of ICT Budget	No	7 (100)	0	0	0	4 (100)	0	0	0
		Yes	0	16 (80)	8 (100)	15 (100)	0	6 (100)	12 (100)	42 (75)
		Missing	0	4 (20)	0	0	0	0	0	14 (25)
Access to Network Connectivity	Access to Internet Services	No	7 (100)	0	8 (100)	0	4 (100)	0	0	0
		Yes	0	18 (90)	0	15 (100)	0	6 (100)	12 (100)	47 (83.9)
		Missing	0	2 (10)	0	0	0	0	0	9 (16.1)
	Type of Internet service	Wireless	0	7 (25)	0	6 (40)	0	1 (16.7)	3 (25)	5 (8.9)
Access to Network Connectivity		Wireless, Modem	0	8 (40)	0	9 (60)	0	0	1 (8.3)	17 (30.4)
		Portable Modem	0	5 (25)	0	0	0	5 (83.3)	2 (16.7)	2 (3.6)
		Wireless, LAN, Modem	0	0	0	0	0	0	0	17 (30.4)
		Missing	0	0	0	0	0	0	6 (50)	15 (26.8)
	Does HF have a web-portal	No	7 (100)	18 (90)	8 (100)	0	0	5 (83.3)	12 (100)	0
		Yes	0	0	0	15 (100)	0	0	0	56 (100)
		Missing	0	2 (10)	0	0	4 (100)	1 (16.7)	0	0
	Is there use of official e-Mail for internal or external communication?	No	6 (87.5)	19 (95)	8 (100)	0	4 (100)	5 (83.3)	12 (100)	0
		Yes	0	0	0	15 (100)	0	0	0	56 (100)
		Missing	1 (14.3)	1 (05)	0	0	0	1 (16.7)	0	0
	Is the local network enabled for both voice and data?	No	6 (87.5)	17 (85)	6 (75)	0	4 (100)	6 (100)	12 (100)	0
		Yes	0	0	0	15 (100)	0	0	0	41 (73.2)
		Missing	1 (14.3)	1 (15)	2 (25)	0	0	0	0	15 (26.8)
	Is licensed anti-virus installed of the computer?	No	6 (87.5)	15 (75)	6 (75)	0	2 (50)	5 (83.3)	11 (91.6)	13 (23.2)
		Yes	0	0	0	14 (93.3)	0	0	0	36 (64.3)
		Missing	1 (14.3)	5 (25)	2 (25)	1 (6.7)	2 (50)	1 (16.7)	1 (8.3)	6 (10.7)
	Is there dedicated IT personnel?	No	5 (71.4)	18 (90)	8 (100)	0	4 (100)	5 (83.3)	12 (100)	32 (57.1)
		Yes	0	0	0	15 (100)	0	0	0	0
		Missing	2 (28.6)	2 (10)	0	0	0	1 (16.7)	0	24 (42.9)

Despite moderate to high telemedicine knowledge, Technology Readiness at HC IVs was poor, and at some individual HC-IVs having an ICT budget, an ICT section, or access to ICT consultancy services did not equate to provision of any Internet related access and services. All RRHs were reported to have ICT budgets, an ICT section, and Internet access. Only one RRH in Western Region, had access to a web-portal, official email services, a network enabled for voice and data, anti-virus protection, and ICT security and therefore could be considered technology ready. The NRH was well provisioned with budget staff, services and security (Table 5).

Significant positive correlations were found between ICT equipment and budget, quality of service (*p* = 0.003), access to Internet connectivity (*p* < 0.001) and between ICT quality of service and access to network connectivity (p < 0.001).

## Discussion

The key findings of this study were that 70% of health professionals surveyed across three levels of HF were either aware of or had used (41%) telemedicine. However, over 40% of HWs at HC-IV and RRH were telemedicine naïve. All doctors who had used telemedicine were impressed with it. ‘Telemedicine user’ and ‘telemedicine aware’ respondents showed core, clinical, and e-learning readiness for telemedicine. Patients were also generally aware of telemedicine but identified barriers to its use. For technology readiness, although all respondents reported knowledge about telemedicine, only the RRH in District 3 and the national referral hospital appeared to be technology ready for telemedicine. The quality of service, equipment and budget for ICT is still a challenge, especially at the lower HF levels.

Relatively few eHealth or telemedicine readiness studies have been conducted in the developing world. Whilst every country is different, with different health systems, burdens of disease, health needs, infrastructure and political agendas, the key findings of such studies are quite similar. For example, in Palestine mHealth approaches were regarded a promising strategy for mental health treatment interventions [21] while an assessment of telemedicine readiness at public health facilities in Addis Ababa, Ethiopia, showed a degree of readiness for telemedicine rating varied from a weak rating for technology readiness to strong rating for Organizational readiness [22].

It is generally acknowledged that eHealth readiness assessment should be undertaken before implementing an eHealth solution [23], in this case telemedicine. A recent review of eHealth readiness frameworks identified 13 papers covering eight readiness domains [13], whilst another systematic review of 63 papers identified 7 differently phrased eHealth readiness domains [14]. There is much confusion and inconsistency in the field of eHealth readiness assessment. No generic framework or underlying unified theory has been reported. Some frameworks address specific issues, with different target audiences assessed, in environments with different degrees of eHealth exposure and experience. Additionally, different tools have been used to score readiness assessment. The review noted that different survey tools will be needed for different groups such as managers, funders, technical staff, doctors, nurses and patients [13]. The tools will also have to differ depending on the proposed eHealth solution, with assessment of readiness for synchronous videoconference based telemedicine being different to that for deployment of a hospital information system.

In part, this study addressed some of these issues. The readiness of three sectors of stakeholders was assessed each using specific tools and methods: health workers at different levels of health facility; management, IT and technical experts; and patients. The large sample size of health workers surveyed (406 participants) provided unusual insight of readiness for telemedicine, primarily because three groups were identified based on their use and / or awareness of telemedicine (telemedicine user, telemedicine aware, and telemedicine naïve), and compared. The responses of those who were unaware of telemedicine prior to the survey (telemedicine naïve) are based solely on their perception, and can be likened to the opinions of participants in a Technology Acceptance Model (TAM) study; TAM refers to an information systems theory that models how users come to accept and use technology, based on their perception of its usefulness and ease of use [24]. The group aware of telemedicine but not using it (telemedicine aware) is also of interest. Although not specifically asked, their reasons for not using telemedicine or their failure to use telemedicine may reflect concerns over legal and regulatory issues, the quality of care provided by telemedicine, or may merely reflect lack of access to technology. Based on their responses the latter is most likely.

Patient readiness for telemedicine was unclear, since patients in this study seemingly equated telehealth to use of the mobile phone. Attempting to understand patient readiness is uncommon but important, because as availability increases it is likely patients, even the general population, will be primary users. Certainly in developed countries the importance of a citizen-centric focus, and empowering citizens as change agents, has been embraced (3). The insight gained from this study will facilitate the development of a tool for determining population readiness.

A recent eHealth readiness study in Mauritius found that 80% of respondents were aware of eHealth or had used telemedicine [25]. Khoja et al.’s eHealth readiness tool was used with managers and healthcare professionals already involved in an eHealth project [11]. In South Africa, the same tool was found to be problematic when used with district managers and hospital managers who were not involved in an eHealth project [26]. Saleh et al. [27], assessed health provider readiness on computer literacy, use, and access to computers at work in the Lebanon. Readiness for eHealth implementation was based on Holt’s Readiness for Organizational Change scale (appropriateness, management support, change efficacy, personal beneficence [28] and showed that eHealth implementation is dependent on readiness of the health providers for change.

All groups (users, aware and naïve) showed a positive attitude for core readiness and integration of telemedicine. Process workflow has been defined as all activities related to patient care and can refer to clinical and administrative workflows and integrated IT solutions are considered critical to optimizing workflows in healthcare [29]. As noted earlier, the user and aware groups averaged over 80% positive responses for all but process workflow, a domain in which low scores for changes to work flow and practice indicate a positive attitude to telemedicine. The respondents showed readiness to integrate telemedicine at the HFs, especially among those who had used telemedicine in the delivery of healthcare.

Both telemedicine users and non-users showed clinical readiness for telemedicine integration at the public health facilities. Health workers see the potential benefits of telemedicine but do not yet have access to the infrastructure necessary to take advantage of it. The willingness to use ICT for health may reflect growing use of social media platforms and comfort with using computers and mobile phones.

This study has further demonstrated that e-learning is being used, and that the majority feel it would be of benefit and are ready to use it. These findings also align with the literature, which provides many examples of the use of e-learning to educate, train, or maintain the skills of the health and social care workforce in both developed (NHS eLearning; https://www.elearning.nhs.uk/), and developing countries [26, 30].

While this study did not comprehensively survey all HF sites, the likelihood is that most facilities will not have adequate infrastructure. While many HWs were either aware of and / or using telemedicine, the HC-IVs lacked infrastructure and the RRH’s lacked secure Internet and Web access for communication, prerequisites for both asynchronous (store and forward) and synchronous (real time) telemedicine. If the necessary technology and user training is not available, successful telemedicine cannot ensue. The introduction of 3G/4G networks offering unlimited Internet data packages [31] presents an opportunity for technology innovations for sites with limited connectivity and bandwidth.

The study also supported the literature which shows not only the need for eHealth readiness assessment, but the need for consistent terminology and description of a limited number of discrete readiness types, and the need for standardised tools for readiness assessment of different user groups (e.g., managers, funders, technical staff, doctors, nurses, patients, policy makers). Future research should focus on the latter two requirements.

Overall, the study findings can guide policy- and decision- makers in the health sector when designing, implementing, and scaling-up telemedicine services in Uganda and similar developing countries. It is recommended that eHealth readiness assessment be conducted for a specific intervention, and for specific groups in a particular healthcare setting using appropriate group specific readiness assessment tools, and not be generalised across all levels of HF. This will ensure evidence-based implementation and integration of telemedicine solutions relevant to the beneficiaries.

### Strength and limitations

A major strength for this paper is that eHealth readiness was addressed using the critical domains and sub-domains identified from the literature as relevant to the Ugandan and developing country context. In addition, this is believed to be the largest and most disparate sample surveyed regarding readiness. Thus the total sample size was 534 participants, and study participants were diverse (ranging from patients, nurses/midwives, district health teams, public health professionals, consultants, doctors and physicians in-charge). Also, both users and non-users of telemedicine services were included in the analysis to ascertain core, clinical and e-learning readiness to integration of telemedicine. Limitations of the study were that the readiness of the private healthcare sector has not been investigated and that respondents’ perceptions of, and understanding of, telemedicine varied.

## Conclusion

With regards to core, clinical and e-learning readiness for integration of telemedicine into public health facilities in Uganda, doctors across all health facility levels are typically more ready than other health professionals. This is particularly so for those who are aware of and have used telemedicine, highlighting the importance of knowledge of and exposure to telemedicine services. Unsurprisingly patients at the varying HF levels, especially the lower level HC-IVs, were sceptical and non-committal to integration of telemedicine, also perhaps because of lack of knowledge and exposure. Of practical concern is the fundamental technology gap in terms of necessary ICT equipment, which requires immediate attention prior to on-going attempts to integrate telemedicine services. This work provides a foundation for further studies on readiness in Uganda and other developing countries concerning implementation of other aspects of eHealth.

## Additional files

Additional file 1: **Appendix 1.** Core, Clinical and eLearning Readiness Assessment Questionnaire. (DOC 66 kb)

Additional file 2: **Appendix 2.** Technology Readiness Assessment Questionnaire. (DOC 69 kb)

Additional file 3: **Appendix 3.** Focus Group Discussion Guide with patients. (DOC 30 kb)

Additional file 4: **Appendix 4.** Detailed breakdown of results by health facility. (DOC 91 kb)

## Abbreviations

FGD: Focus Group Discussion
HC-IVs: Health Center Fours
HFs: Health Facilities
HWs: Health Workers
ICT: Information and Communications Technology
IT: Information Technology
NRH: National Referral Hospital
OPD: Out-Patient Department
RRH: Regional Referral Hospital
WHO: World Health Organization
