# Supplementary material for: Assessing core, e-learning, clinical and technology readiness to integrate telemedicine at public health facilities in Uganda: a health facility – based survey
Source: BMC Health Serv Res. 2019 Apr 29;19:266. doi: 10.1186/s12913-019-4057-6 (PMC6489273; doi:10.1186/s12913-019-4057-6)
Supplement: Supplementary file 1 — Appendix 1. Core, Clinical and eLearning Readiness Assessment Questionnaire. (DOC 66 kb) [file 12913_2019_4057_MOESM1_ESM.doc]

|  | **Response** | **HC-IV (N=15)** | | **RRH (N=71)** | | **NRH (N=81)** | | **Total** | | **p =** |
| --- | --- | --- | --- | --- | --- | --- | --- | --- | --- | --- |
|  |  | **Doctors**  **(n=3)** | **Other**  **(n=12)** | **Doctors**  **(n=29)** | **Other**  **(n=42)** | **Doctors**  **(n=42)** | **Other**  **(n=39)** | **Doctors**  **(n =74)** | **Others**  **(n=93)** |  |
| **TM used for** | Diagnosis | 0 | 1 (33.3) | 2 (6.9) | 0 | 14 (33.3) | 8 (20.5) | 16 (34.0) | 9 (9.7) | 0.032 |
|  | Treatment | 2 (66.7) | 1 (33.3) | 2 (6.9) | 4 (9.5) | 19 (45.2) | 9 (23.1) | 23 (31.1) | 14 (15.2) | 0.013 |
|  | Prevention | 3 (100) | 1 (33.3) | 1 (3.4) | 2 (4.8) | 21 (50) | 12 (30.8) | 25 (33.8) | 15 (16.1) | 0.008 |
|  | e-Learning | 1 (33.3) | 3 (100) | 19 (65.5) | 27 (64.3) | 10 (23.8) | 26 (66.7) | 30 (40.5) | 40 (43.0) | NS |
|  | Knowledge sharing | 1 (33.3) | 1 (33.3) | 9 (31) | 16 (38.1) | 11 (26.2) | 14 (35.9) | 21 (28.4) | 31 (33.3) | NS |
|  | e-Consultation | 1 (33.3) | 3 (100) | 22 (75.9) | 21 (50) | 24 (57.1) | 22 (56.4) | 47 (63.5) | 46 (49.5) | NS |
|  | e-Prescription | 0 | 0 | 20 (69) | 1 (2.4) | 40 (95.2) | 0 | 60 (81.1) | 1 (1.1) | 0.001 |
| **Impressed with TM** | Yes | 3 (100) | 5 (41.7) | 29 (100) | 37 (88.1) | 42 (100) | 39 (100) | 74 (100) | 81 (87.1) | 0.001 |
|  | No | 0 | 7 (58.3) | 0 | 5 (11.9) | 0 | 0 | 0 | 12 (12.9) |  |
| **TM reduces referrals** | Yes | 3 (100) | 8 (66.7) | 21 (72.4) | 32 (76.2) | 41 (97.6) | 33 (84.6) | 65 (87.8) | 73 (78.5) | NS |
|  | No | 0 | 3 (25) | 4 (13.8) | 2 (4.8) | 1 (2.4) | 4 (10.3) | 5 (6.8) | 9 (9.7) |  |
|  | Missing | 0 | 1 (8.3) | 4 (13.8) | 8 (19.0) | 0 | 2 (5.1) | 4 (5.4) | 11 (11.8) |  |
| **TM decongests hospital** | Agree | 3 (100) | 12 (100) | 24 (82.8) | 33 (78.6) | 38 (90.5) | 15 (38.5) | 65 (87.8) | 60 (64.5) | 0.001 |
|  | Disagree | 0 | 0 | 4 (13.8) | 2 (4.8) | 0 | 0 | 4 (5.4) | 2 (2.2) |  |
|  | Not sure | 0 | 0 | 1 (3.4) | 7 (16.7) | 4 (9.5) | 24 (61.5) | 5 (6.8) | 31 (33.3) |  |
| **Use TM vs Traditional** | Yes | 3 (100) | 11 (91.7) | 25 (86.2) | 22 (52.4) | 39 (92.9) | 36 (92.3) | 67 (90.5) | 69 (74.2) | 0.007 |
|  | No | 0 | 1 (8.3) | 2 (6.9) | 10 (23.8) | 3 (7.1) | 3 (7.7) | 5 (6.8) | 14 (15.1) |  |
|  | Missing | 0 | 0 | 2 (6.9) | 10 (23.8) | 0 | 0 | 2 (2.7) | 10 (10.8) |  |
| **Required**  **before TM implemented** | Licensing | 2 (66.7) | 12 (100) | 12 (41.4) | 13 (31) | 29 (69) | 39 (100) | 43 (58.1) | 64 (68.8) | NS |
|  | Remuneration | 0 | 4 (33.3) | 14 (48.3) | 9 (21.4) | 19 (45.2) | 39 (100) | 33 (44.6) | 52 (55.9) | NS |
|  | Policy | 3 (100) | 12 (100) | 29 (100) | 29 (69.0) | 42 (100) | 39 (100) | 74 (100) | 80 (86) | 0.001 |
|  | Training | 3 (100) | 12 (100) | 29 (100) | 29 (69.0) | 36 (85.7) | 38 (97.4) | 68 (91.9) | 79 (84.9) | NS |
|  | Ethical Guideline | 3 (100) | 12 (100) | 29 (100) | 19 (45.2) | 37 (88.1) | 30 (76.9) | 69 (93.2) | 61 (65.6) | 0.001 |
| **TM solves**  **HWs crisis** | Yes | 3 (100) | 6 (50) | 25 (86.2) | 34 (81) | 42 (100) | 34 (87.2) | 70 (94.6) | 74 (79.6) | 0.005 |
|  | No | 0 | 6 (50) | 4 (13.8) | 8 (19) | 0 | 5 (12.8) | 4 (5.4) | 19 (20.4) |  |
| **Is TM cost effective?** | Agree | 3 (100) | 12 (100) | 23 (79.3) | 42 (100) | 33 (78.6) | 39 (100) | 59 (79.7) | 93 (100) | 0.001 |
|  | Disagree | 0 | 0 | 6 (20.7) | 0 | 9 (21.4) | 0 | 15 (20.3) | 0 |  |
| **Is it worth investing in infrastructure?** | Yes | 3 (100) | 10 (83.3) | 29 (100) | 32 (76.2) | 41 (97.6) | 34 (87.2) | 73 (98.6) | 76 (81.7) | 0.001 |
|  | No | 0 | 2 (16.7) | 0 | 6 (14.3) | 0 | 3 (7.7) | 0 | 11 (11.8) |  |
|  | Missing | 0 | 0 | 0 | 4 (9.5) | 1 (2.4) | 2 (5.1) | 1 (1.4) | 6 (6.5) |  |
