# Supplementary material for: Assessing core, e-learning, clinical and technology readiness to integrate telemedicine at public health facilities in Uganda: a health facility – based survey
Source: BMC Health Serv Res. 2019 Apr 29;19:266. doi: 10.1186/s12913-019-4057-6 (PMC6489273; doi:10.1186/s12913-019-4057-6)
Supplement: Supplementary file 2 — Appendix 2. Technology Readiness Assessment Questionnaire. (DOC 69 kb) [file 12913_2019_4057_MOESM2_ESM.doc]

| **No#** | **QUESTIONS AND FILTERS** | **CODING CATEGORIES** | **SKIP** | |
| --- | --- | --- | --- | --- |
| **SECTION 1. RESPONDENT’S BACKGROUND** | | | | |
| 1 | What is your current job title? | Medical Doctor …………………………...1  Surgeon …………………………………...2  Midwife/Nurse …………………................3  Pharmacist ……………………..…….…...4  Radiographer …………………………......5  OTHERS (Specify) ……………………...6 |  | |
| 2 | What level of health facility are you working with? | National Referral…………………...…......1  Regional Referral……………………….....2  General Hospital………………………......3  Health Center IV………………………......4 |  | |
| 3 | For how long have you worked in your current position? | 0-2 y………………………………….........1  3-5 y……………………………….…........2  6-10 y………………………………...........3  11-15 y……………………………….........4  16-20 y……………………………….........5  Others (Specify)…………..………….........6 |  | |
| 4 | How many are you in your field of specialization working at this healthcare institution? | Alone ………………….……………….....1  2-3……………………………………........2  4-5………………………………………....3  >5……………………………………….....4  Don’t know…………..…………………....5 |  | |
| 5 | What is your highest education level that you have attained? | Certificate ………………………………...1  Diploma ………………………....……......2  Advanced Diploma……………………......3  Degree……………..……………………....4  Masters………………………………….....5  Doctorate……………………………….....6  Others (Specify) ………………………......7 |  | |
| 1. **CORE READINESS ASSESSMENT** | | | | |
| **AWARENESS OF TELEMEDICINE** | | | | |
| 6 | Do you know what Telemedicine means? | Yes..……………….……………………....1  No………………………………………....2 | 11 | |
| 7 | Have you ever used Telemedicine? | Yes...……………………………………....1  No………………………………………....2 |  | |
| 8 | What did you use it for? | Diagnosis……………..…………………...1  Treatment……………………….................2  Prevention……………...……………….....3  e-learning…………………..……………...4  Knowledge sharing………...……………...5  e-consultation……………………...............6  Others (Specify)…………………………...7 | Tick whatever applies. | |
| 9 | Were you impressed by the Telemedicine service? | Yes………………..…………………….....1  No……..………………………………......2 |  | |
|  |  | NEEDS ASSESSMENT |  | |
| 10 | On average, about how many patients do you attend to in a day? | 0-10…………………………...…………...1  11-20…………………………...……….....2  21-30….……………………...…………....3  31-40…………………………...……….....4  41-50…………………………...……….....5  >50……………………………..……….....6 |  | |
| 11 | Of those you meet in a day, how many do you recommend for a referral? | 1-5……………..……………..…………....1  6-10…………………………..…………....2  >10……………………………..……….....3  None……………………………..………...4  Others (Specify)……………………...…....5 |  | |
| 12 | Where do you often refer your patients? | General Hospital……………………...…...1  Regional Referral Hospital……………......2  National Referral Hospital………...............3  Overseas…………………………………...4 |  | |
| 13 | Usually under what circumstances do you choose to refer your patients? | Shortage of drugs….…….………………...1  Shortage of equipment.……….…………...2  Consultation required…………….…….....3  Special investigation required ……….…...4  All the above…………..……………..........5  None of the above………………................6 |  | |
| 14 | Who often initiates the referral? | Patient/Family caretaker……….……….....1  Healthcare provider……………….……....2  Others (Specify) ………..………………....3 |  | |
|  |  | INTEGRATING TELEMEDICINE |  | |
| 15 | Do you think use of Telemedicine will minimize referrals? | Yes………………………………………...1  No………………………………………....2 |  | |
| 16 | Would you think use of Telemedicine service will decongest referral hospitals? | Disagree…………………………..….........1  Agree……………………………...............2  Strongly Agree…………..………………...3  Not very sure……………………………...4  May be with time………………………....5 |  | |
| 17 | Would you prefer using Telemedicine to traditional mode of care? | Yes………………………………………...1  No…………….…………………………...2 |  | |
| 18 | What needs to be done for you as a medical worker before start use of Telemedicine? | Training…………………………………...1  Licensing…………………………….........2  Remuneration……………………………..3  Policy……………………………………...4  Ethical guidelines………………………....5  Others (Specify)……………………….......6 |  | |
| 19 | As a medical worker, would you consider using telemedicine without prior physical interaction with the patient? | Disagree…………………………………...1  Agree………….…………………………..2  Strongly Agree……………..……………..3  Not Sure…………………………………..4  May be with time……………………..…..5  Others (Specify)…………………..............6 |  | |
| 20 | Could Telemedicine be an answer to current HW’s crisis or a threat to HW’s positions? | Yes.……………..……………………........1  No………………………………………....2 |  | |
| **COMFORT USING TELEMEDICINE** | | | | |
| 21 | In your perception, is use of telemedicine cost effective compared to traditional mode of care? | Disagree…………..………………................1  Agree………………..…………………........2  Strongly Agree………………………............3  Not Very sure………………………….........4  May be with time………………………........5 | |  |
| 22 | Is telemedicine worth an investment to establish the required infrastructure? | Disagree…………………………………......1  Agree……………………………………......2  Strongly Agree…………………………........3  Not very sure……………………………......4  May be with time………………………........5 | |  |
| 23 | How about in terms of cost to training HWs to use Telemedicine services? | Yes..……………………………………........1  No.………………………………………......2 | |  |
| 24 | Do you see Telemedicine an effective service in cases of emergencies? | Yes………………………………………......1  No...……………………………………........2 | |  |
| **PROCESS WORKFLOW** | | | | |
| 25 | Do you think telemedicine services would affect normal process workflow at a health facility? | Disagree…………………………………......1  Agree………...………..……………….........2  Strongly Agree…...…………..………….......3  Not very sure………..……..……………......4  May be with time……….....…………….......5 | |  |
| 26 | Do you see integration of telemedicine services alter existing work practices at the health facility? | Yes..………………………………….….......1  No.………………………………………......2 | |  |
| 27 | Would use of telemedicine services challenge physician’s referral methods? | Disagree……………………...………….......1  Agree…………………………..………........2  Strongly Agree…………………...…….........3  Not very sure……………………...…….......4  May be with time………………………........5 | |  |
| 28 | In your view, do you think HWs are ready to use telemedicine services for medical practice? | Yes...……………..…………………….........1  No.………………………………………......2 | |  |
| 1. **LEARNING READINESS** | | | | |
|  |  | **ENHANCING PROVIDER’S SKILLS** | |  |
| 29 | How do you usually aim at CME? | Attend Workshops………………………......1  Conferences……..………………………......2  Attend a Course ……………………….........3  Use the Internet………….…….…….............4  Consult colleagues …………..…...................5  None of the above ………………..................6 | |  |
| 30 | Do you think introducing telemedicine will facilitate e-learning to enhance health providers’ education? | Disagree……………………………..…........1  Agree…………………………………..........2  Strongly Agree……………………..…..........3  Not very sure…………………………..........4  May be with time………………………........5 | |  |
| 31 | Are health providers ready to adopt e-learning to acquire additional skills? | Yes...…………………………………….......1  No.….……………….…………………........2 | |  |
| 32 | Would Telemedicine enable remote consultations/knowledge sharing amongst health providers? | Yes...…………………..……………….........1  No..…………………………………….........2 | |  |
| 33 | Would knowledge sharing bridge skills gap with health providers? | Yes..……………………………………........1  No.……………..………………………........2 | |  |
| 1. **HWs READINES FOR CLINICAL TELEMEDICINE** | | | | |
| 34 | Would you consider use of telemedicine services for clinical care? | Yes………………………………………......1  No...……………….…………………….......2 | |  |
| 35 | Would you agree with patients’ outcomes as a result of e-prescription and/or e-consultation? | Yes………………..……………….…….......1  No...……………………………………........2 | |  |
| 36 | Do you see telemedicine as a means to improve patients’ outcome through enabling access to specialized clinical care? | Yes………………………………………......1  No……………..……………..………...........2 | |  |
