# Supplementary material for: Assessing core, e-learning, clinical and technology readiness to integrate telemedicine at public health facilities in Uganda: a health facility – based survey
Source: BMC Health Serv Res. 2019 Apr 29;19:266. doi: 10.1186/s12913-019-4057-6 (PMC6489273; doi:10.1186/s12913-019-4057-6)
Supplement: Supplementary file 3 — Appendix 3. Focus Group Discussion Guide with patients. (DOC 30 kb) [file 12913_2019_4057_MOESM3_ESM.doc]

**Awareness about Telemedicine**

1. What does telemedicine mean to you? **Probe**:
   1. Do you know what telemedicine is?

**Comfort using Telemedicine**

1. Have you ever used telemedicine to seek or receive medical care?

**If YES,**

1. What was your experience like when you used telemedicine?
2. What prompted the use of telemedicine other than seeking or receiving care at the near by healthcare facility (HF)?
3. Were you satisfied by the medical care received while using telemedicine?
4. How would you compare it with the traditional mode of healthcare?
5. What is your recommendation after such an experience?

**If NOT,**

1. Under what circumstances would telemedicine compromise privacy to your personal medical details?
2. Would you feel convinced with the quality of care delivered at a distance without physical contact with the physician?

**Integrating Telemedicine**

1. Do you see use of telemedicine services an effective alternative means to healthcare delivery or an inconvenience to the health worker force?
2. Should Uganda’s health sector invest money into integrating telemedicine within it’s healthcare system?
3. Do you see integrating telemedicine services a solution to cases of health emergencies?
4. Would you prefer to use telemedicine services to seek or receive medical care compared to a referral to a higher HF level to access quality healthcare?
5. What would be the possible barriers to effectiveness and efficiency in using telemedicine at the health facility?

Other than what we have already discussed, is there anything you would like to share that will benefit our study?

**THANK YOU SO MUCH**
