# Supplementary material for: Assessing core, e-learning, clinical and technology readiness to integrate telemedicine at public health facilities in Uganda: a health facility – based survey
Source: BMC Health Serv Res. 2019 Apr 29;19:266. doi: 10.1186/s12913-019-4057-6 (PMC6489273; doi:10.1186/s12913-019-4057-6)
Supplement: Supplementary file 4 — Appendix 4. Detailed breakdown of results by health facility. (DOC 91 kb) [file 12913_2019_4057_MOESM4_ESM.doc]

| NO# | **QUESTIONS AND FILTERS** | | **CODING CATEGORIES** | **SKIP** |
| --- | --- | --- | --- | --- |
| **GENERAL QUESTIONS** | | | | |
| 1 | Do you know what Telemedicine means? | | Yes…………….……………………........1  No…………….………………........…….2 | 5 |
| 2 | Have you ever used Telemedicine? | | Yes……………..…....…………………...1  No…………….………....……………….2 | 5 |
| 3 | What did you use it for? | | Diagnosis….………….........…………….1  Treatment…………...…............................2  Prevention……………..............…………3  e-learning…..…..….............……….…….4  Knowledge sharing...…………............….5  e-consultation……..…………..................6  Others (Specify)…................................….7 |  |
| 4 | Were you impressed by the Telemedicine service? | | Yes……………..….................………..…1  No……………...............………………...2 |  |
| SECTION 1. **RESPONDENT’S BACKGROUND** | | | | |
| 5 | What level is your healthcare institution and/or organization? | | NRH……………..............……………….1  RRH…………….…..................................2  GH……………….....................................3  HCIV…………….........…........................4  District Health Office (DHO)…...……….5 |  |
| 6 | What is your position/role at your health institution/organization? | | Executive Director……..........…………...1  Administrator….............…………………2  Head of Section………….........................3  Health In-Charge………….................…..4  District Health Officer………..….............5  Others (Specify)………...................……..6 |  |
| 7 | For how long have you worked in that position? | | 0-1yr……………...........…………………1  1>=3yr…….…........…………………….2  3>=5 yrs………..............………………...3  5>=8 yrs……………….............…………4  8>=10 yrs………………..................…….5  10>=15 yrs…………..……...............……6  Others (Specify)………...................……..7 |  |
| **ICT TECHNICAL PERSONNEL** | | | | |
| 8 | Do you have an ICT section in your health institution? | | YES……………...............……………….1  NO…………...........……………………..2 | 11 |
| 9 | What is the human resource capacity in the ICT section? | | 0-5…. …………………............................1  6-10..…………….............……………….2  Others (Specify)……….................………3 |  |
| 10 | What level of education are your ICT staffs? | | Diploma………...............………………..1  Degree…………..............………………..2  Masters……….................………………..3  Others (Specify)……….................………4 |  |
| 11 | Do you have access to ICT expert consultants? | | YES…………….............….......................1  NO………….…...........………………….2 |  |
| 12 | What proportion of your staff knows how to use a computer? | | ALL…………............…………………...1  75%............................................................2  50%............................................................3  25%............................................................4  Others (Specify)…….....…........................5 |  |
|  |  | **EXISTING ICT EQUIPMENT & BUDGET** | |  |
| 13 | What are the available ICT infrastructures? | | Desktops…………........…………………1  Servers…………....…..………………….2  Monitor……………........………………..3  Phones……….......………………………4  Laptops..…….........……….......................5  Photocopier……........………….………...6  Document scanner…………...........……..7  TV-based conferencing ……….………...8  PC-based conferencing………….............9  Web cam connected…………….………10  Digitalized X-Ray equipment....………..11  High resolution digital Cam monitored on a microphone………...............................12  Others (Specify)…………….........……..13 | **Circle whatever applies.** |
| 14 | Do you have an ICT budget for your institution? | | Yes……………………....…………….....1  No………………………...……………...2 | 17 |
| 15 | What is the budget range per year? | | 0-50 Millions……………..............……...1  50-100 Millions…………………....…….2  100-500 Millions………………...............3  500-700 Millions………….............……..4  >700 Millions……………..........………..5  Others (Specify)…………….....................6 |  |
| 16 | Who funds your ICT budget? | | Donors……………...........………………1  Government………...........……................2  Internally…………....................................3  Others (Specify)………….............………4 |  |
| **ACCESS TO NETWORK CONNECTIVITY** | | | | |
| 17 | Do your health providers have access to Internet services at the health facilities? | | YES………………........…………………1  NO………………........………………….2 | 20 |
| 18 | What is your Internet service? | | Wireless………............………………….1  ADSL………….............…….….….…….2  Dial-up……………...............……………3  Others (Specify)……….............…………4 |  |
| 19 | Does your healthcare facility have a web-portal? | | Yes……………............………………….1  No....…………....………………………..2 |  |
| 20 | Do your staff use official emails for any internal or external communication? | | Yes……………....……………………….1  No………………....……………………..2 | 22 |
| 21 | Where do your staff access email services from? | | Office Computer...………............……….1  Personal Phones……..................………...2  Home Computer….…............…………...3  Private Cafes…………..............…………4  Others (Specify)……….............…..……..5 |  |
| 22 | What is the other form of communication used at your healthcare institution? | | Fixed Phone………...........……………....1  Faxing……………......…………………..2  Video Conferencing….…..............………3  Skyping……….............….........................4  Others (Specify)……..............……….......5 |  |
| 23 | Is your network enabled for both data and voice transfers? | | Yes………............…………………….…1  No……….....…………………………….2 |  |
| 24 | Is it a LAN/WAN? | | LAN………..........……………………….1  WAN…………......………………………2 |  |
|  |  | | **ICT QUALITY OF SERVICE** |  |
| 25 | What is the computer per personnel ratio? | | 1:5 ……………...........…………………..1  1:10………….......……………………….2  1:15………….......……………………….3  1:20…………….......…………………….4  Others (Specify)……...........……………..5 |  |
| 26 | What is your current Internet bandwidth? | | <=1Mgb/sec…................………………...1  1<=3 Mgb/sec……..........………………..2  3<=5 Mgb/sec………...................……….3  >5Mgb/sec……............…….....................4  Others (Specify)……….................………5 |  |
| 27 | What is the nature of your bandwidth? | | Dedicated…….................………………..1  Shared…………...........………………….2  Others (Specify)………............………….3 |  |
| 28 | What proportions of your computers have full time connection to Internet? | | All…………….......……………………...1  75%............................................................2  50%............................................................3  25%............................................................4  None…………........……………………...5  Others (Specify)………...................……..6 |  |
|  |  | | **IT SECURITY** |  |
| 29 | What security tools do you have on your network? | | Firewall System………….........…………1  Network VPN……………........................2  Anti-virus software………………………3  Access Control Levels….………………..4  Others (Specify)………….........................5 |  |
| 30 | Do you have a licensed anti-virus installed on your computers? | | Yes………..........…………………….......1  No…………........………………………..2 |  |
| 31 | Do you have a dedicated staff who manages IT security? | | Yes…………........……………………….1  No...………..…………………………….2 |  |
| 32 | Who helps you solve security breaches on the network? | | Consultant…….............………………….1  None………..........……………………….2 |  |
